# Objective Analyses of Tessellated Fundi and Significant Correlation between Degree of Tessellation and Choroidal Thickness in Healthy Eyes

**DOI:** 10.1371/journal.pone.0103586

**Published:** 2014-07-28

**Authors:** Naoya Yoshihara, Takehiro Yamashita, Kyoko Ohno-Matsui, Taiji Sakamoto

**Affiliations:** 1 Department of Ophthalmology, Kagoshima University Graduate School of Medical and Dental Sciences, Kagoshima, Japan; 2 Department of Ophthalmology and Vision Science, Tokyo Medical and Dental University, Tokyo, Japan; Massachusetts Eye & Ear Infirmary, Harvard Medical School, United States of America

## Abstract

A tessellated fundus is a common characteristic of myopic eyes and is an important clinical marker for the development of retinochoroidal changes. However, the exact cause and significance of tessellated fundi have not been definitively determined. We determined the degree of tessellation in fundi objectively in normal, non-pathological myopic eyes, and correlated the degree of tessellation and the choroidal thickness (CT) and axial length (AL). This was a prospective observational cross sectional study. The eyes were classified subjectively into three groups based on the degree of tessellation observed ophthalmoscopically. Digital color fundus photographs were assessed for the degree of tessellation by ImageJ, an image processing program. Three tessellated fundus indices (TFIs) were calculated and were compared to the three subjectively-determined groups. The subfoveal and nasal CTs were measured in the optical coherence tomographic images. The correlations between the TFIs and the CT were calculated. Additionally, the correlation between the TFIs and the AL was calculated. One hundred right eyes of 100 healthy volunteers (mean age 25.8±3.9 years) were studied. Ophthalmoscopically, 57 eyes were placed in the non-tessellated group, 27 eyes into the weakly tessellated group, and 16 eyes into the strongly tessellated group. There was a significant correlation between the subjective classifications and the TFI values (*P*<0.05, Kruskal-Wallis test). All of the TFIs were significantly associated with the subfoveal and nasal CT (R = −0.20 to −0.24, *P*<0.05). The TFIs were not significantly correlated with the ALs. In conclusion, the significant correlation between the subjective and objective classifications of the degree of tessellation indicates that TFIs can be used to classify the degree of tessellation. The results indicate that the differences in the CT account for the degree of tessellation.

## Introduction

High myopia is a common cause of blindness in the Western countries [1], and it is a more serious problem in Asian countries because it is much more prevalent and the degree of myopia is higher [2]. Thus, myopic maculopathy was reported to be the second most frequent cause of blindness in China and third most frequent cause in Japan [2], [3]. Additionally, the Tajimi study in Japan found that the incidence of myopia in the Japanese population was the highest in the world [4].

The problem of evaluating highly myopic eyes is because of their wide clinical variations. High myopia is usually defined as those eyes with a refractive error of ≥−6.0 diopter, however myopic retinochoroidal atrophies do not occur in all eyes with these higher refractive errors. Similarly, myopic retinochoroidal atrophy often occurs in myopic eyes with longer axial lengths but not in all of these eyes [5]. A tessellated fundus is a common characteristic of myopic eyes, and has been found to be an important clinical marker for the development of retinochoroidal changes [6], [7]. However, this important clinical finding is not easy to be assessed because there is no objective method to grade the degree of tessellation. Therefore, an objective and quantitative method for assessing a tessellated fundus is needed so that it can be used for clinical and experimental studies.

Earlier, Suzuki used a color imaging software to analyze digital fundus images of eyes with Vogt-Koyanagi-Harada (VKH) disease to obtain a “sunset glow index” [8]. They reported that the sunset grow index determined objectively with this software was significantly correlated with the duration of the disease and a decrease in the number of melanocytes. They concluded that their objective method can be used to evaluate the degree of activity of VKH disease [8]. More recently, Neelum et al used a similar method and quantified the degree of myopic chorioretinal degeneration. They reported that the degree of retinochoroidal degeneration was correlated with the axial length of the eye and the age [9]. However, they did not evaluate the choroidal thickness (CT) which is believed to be an important parameter in evaluating choroidal degeneration.

Evaluations of the morphology of the posterior pole of the eye have been greatly improved by the advancement of optical coherence tomography (OCT). Thus, Spaide et al developed a technique of enhanced depth imaging (EDI)-OCT to examine the choroid in greater detail [10]. This technique has enabled clinicians to examine the choroidal structures more accurately [10], and to study a large number of eyes *in situ* in a relatively short time.

Studies of the choroid by EDI-OCT showed that the choroid of highly myopic eyes was significantly thinner than that of normal eyes [11]–[14]. Nonetheless, to the best of our knowledge, there has not been a study published that determined whether there was a significant correlation between the degree of tessellation and the CT and also with other morphological parameters of the eye.

Thus, the purpose of this study was to determine and validate an objective method of quantifying the degree of tessellation of the ocular fundus. To accomplish this, we classified the degree of tessellation subjectively by the ophthalmoscopic appearance, and then classified the digital color fundus photographs of the same eyes with a color image processing software, ImageJ (version 1.47; National Institutes of Health, Bethesda, MD, USA; http://imagej.nih.gov/ij/[in the public domain]). We shall show that there were significant correlations between the subjective and objective degrees of tessellation of the fundus. We also examined whether the degree of tessellation determined objectively was significantly correlated with the CT and axial length of the eye.

## Methods

### Ethics statement

All of the procedures used conformed to the tenets of the Declaration of Helsinki. A written informed consent was obtained from all of the subjects after an explanation of the procedures to be used. The study was approved by the Ethics Committee of Kagoshima University Hospital, and it was registered with the University Hospital Medical Network (UMIN)-clinical trials registry. The registration title was, “Morphological analysis of the optic disc and the retinal nerve fiber in myopic eyes” and the registration number was UMIN000006040. A detailed protocol is available at https://upload.umin.ac.jp/cgi-open-bin/ctr/ctr.cgi?function=brows&action=brows&type=summary&recptno=R000007154&language=J.

### Subjects

This was a cross sectional, prospective observational study. One hundred and nine eyes of 109 volunteers were initially studied between November 1, 2010 and February 20, 2012. Volunteers with no known eye diseases as determined by examining their medical records were studied, and only the data from the right eyes were analyzed. The eligibility criteria were: age ≥20 years but ≤40 years; eyes normal by slit-lamp biomicroscopy, ophthalmoscopy, and OCT; best-corrected visual acuity (BCVA) ≤0.1 logarithm of the minimum angle of resolution (logMAR) units; and intraocular pressure (IOP) ≤21 mmHg. The exclusion criteria were: eyes with known ocular diseases such as glaucoma, staphyloma, and optic disc anomaly; known systemic diseases such as hypertension and diabetes; presence of visual field defects; and history of refractive or intraocular surgery. Nine subjects were excluded; 3 eyes with superior segmental optic disc hypoplasia, 1 eye with glaucoma, 3 eyes with previous laser eye surgery, and 2 eyes with incomplete examination. The data from some of the participants of our earlier study were included in this study [15].

### Examinations of eyes

All of the eyes had a standard ocular examination including slit-lamp biomicroscopy of the anterior segment, ophthalmoscopy of the ocular fundus, IOP measurements with a pneumotonometer (CT-80, Topcon, Tokyo, Japan), and axial length measurements with the AL-2000 ultrasound instrument (TOMEY, Nagoya, Japan). In addition, the refractive error (spherical equivalent) was measured with the Topcon KR8800 autorefractometer/keratometer.

### Measurement of CT

The subjects were examined with the Heidelberg Spectralis-OCT (Spectralis; Heidelberg Engineering, Heidelberg, Germany), and the choroidal images were obtained according to a method described in detail [16], [17]. The subfoveal CT was determined as the perpendicular distance from the outer edge of the hyperreflective RPE line to the inner scleral border with a built-in linear measuring tool. In addition, the CT at 750 µm nasal to the fovea was also determined. All images were taken between 15∶00 and 17∶00 hours to avoid possible diurnal variations in the CT [18].

### Quantitative analysis of digital color fundus photographs

The fundi of all subjects were photographed by a single examiner using the same digital fundus camera with standardized settings (TRC-50LX, Topcon, Tokyo, Japan). The digital images were displayed on a computer screen, and they were classified into three groups; non-tessellated group, weakly tessellated group, and strongly tessellated group by 3 experienced retina specialists (HK, NY, MS). The classifications were made independently, and the graders were masked to the digital findings. The final classification was determined by an agreement of at least two of the graders. No case was classified into three different categories.

**Figure 1:**
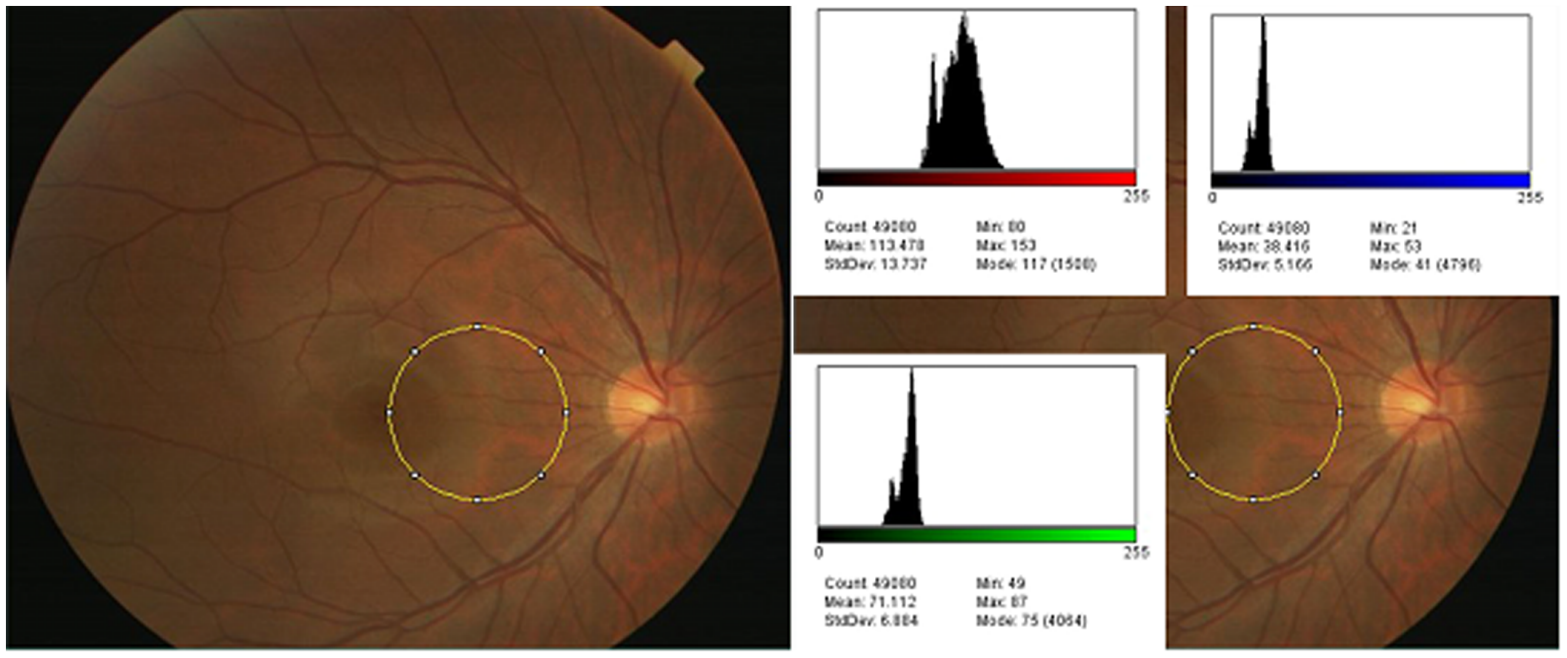
Quantitative analysis of digital color fundus photographs. An area between the fovea and the optic disc was selected as shown by the yellow circle (left). The same color fundus photograph showing the red, green and blue pixels using image J (right). In the histograms, the horizontal bar indicates the brightness of each color. Note that the vertical scale is different for the different histograms. Below each histogram, the mean, standard deviation, minimum, maximum, median, and count for each color are shown.

The digital color fundus photographs were also quantified to a “tessellated fundus index” (TFI) by a method developed in our laboratory. A circular area of 250 pixel diameter between the fovea and the optic disc was first selected to determine the TFI (Fig. 1, left). Our preliminary study showed that the tessellated changes were most prominent in the area around the optic nerve. However, the present color analysis program is likely to be affected by the presence of large retinal vessels and optic disc conus. Thus, the area was determined to be the fovea-macula area. The size, 250 pixels, was also determined by the results of our preliminary study that the examination area should be large as possible but to not include areas with the large retinal vessels and the optic nerve conus.

Next, the ImageJ software (National Institutes of Health, Bethesda, MD; available at: http://imagej.nih.gov/ij/) was used to identify and count the number of R, G, and B pixels within the circle. This was followed by the construction of histograms of the number of red, green, and blue pixels in the circular area (Fig. 1, right).

Earlier studies quantified the fundus color in eyes with myopia, sunset glow fundus of the VKH disease, and concentration of hemoglobin on the optic disc. In tessellated fundi, the fundus color is redder similar to the sunset glow fundus of VKH disease. Therefore, we selected the TFI calculation algorithms from these previous studies [8], [9]. Three TFIs, called TFI-1, TFI-2, and TFI-3, were calculated using the mean red intensity (R), the mean green intensity (G), and the mean blue intensity (B) as follows:
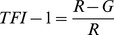





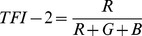





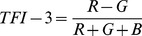



### Statistical analyses

The intra-rater correlation coefficients of the TFIs were calculated using a two-way mixed-effects model for measurements of absolute agreement. The inter-rater agreement of the subjective classifications was assessed with the Fleiss kappa method. The Steel-Dwass multiple comparison test was used to analyze the significant differences of TFIs among the three groups; non- tessellated group, weakly tessellated group, and strongly tessellated group. The relationship between TFI-1, TFI-2, and TFI-3 and the subfoveal and nasal CTs and the axial length was determined by Spearman’s rank coefficients of correlation. All statistical analyses were performed with the statistical programming language R (version 3.0.2, The R Foundation for Statistical Computing, Vienna, Austria). A *P* value of <0.05 was taken to be statistically significant.

## Results

We studied 100 right eyes of 100 individuals. The mean and standard deviation of the age was 25.8±3.9 years with a range from 22.0 to 39.0 years, and 66 were men (66%). The mean and standard deviation of axial length was 25.3±1.4 mm with a range from 22.4 to 30.4 mm. The mean and standard deviation of the refractive error (spherical equivalent) was −4.6±3.3 D with a range from −13.0 to 0.0 D. The mean and standard deviation of the subfoveal CT was 261.8±84.9 µm and the mean nasal CT was 244.0±81.4 µm.

The means and standard deviations of the TFI-1, TFI-2, and TFI-3 were 0.354±0.051, 0.503±0.027, and 0.179±0.033, respectively (Table 1).

**Table 1:** Summary of Clinical Data of All Subjects.

	Mean ± SD	Range
**Age (years)**	25.8±3.9	22.0–39.0
**AL (mm)**	25.3±1.4	22.4–30.4
**SE (diopters)**	−4.6±3.3	−13.0–0.0
**SFCT (µm)**	261.8±84.9	76.0–500.0
**NCT (µm)**	244.0±81.4	76.0–460.0
**TFI-1**	0.354±0.051	0.257–0.533
**TFI-2**	0.503±0.027	0.448–0.595
**TFI-3**	0.179±0.033	0.121–0.317

AL = axial length; SE = spherical equivalent; SFCT = subfoveal choroidal thickness; NCT = nasal of subfoveal choroidal thickness; TFI = tessellated fundus index.

### Intersession correlations of TFIs

Thirty eyes were randomly selected, and the TFIs were determined two times by the same rater (NY). The values were almost perfectly matched between sessions (Figure S1).

### Comparison among three subjective tessellated fundus classifications for TFIs

**Figure 2:**
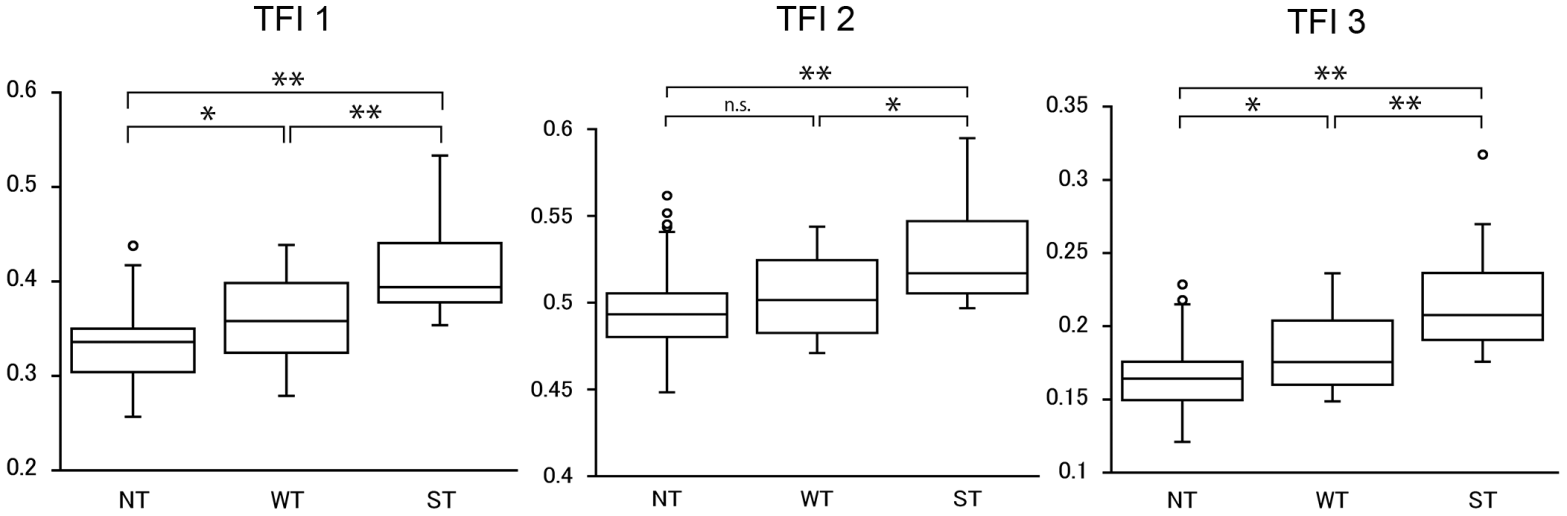
Box plot of subjective classification of tesselation and the TFI values of TFI-1, TFI-2 and TFI-3. For each TFI, the eyes in the strongly tessellated group (ST) had a significantly higher TFI values than those in the non-tessellated group (NT). Those in weakly tessellated group (WT) were in between. The only exception was NT and WT in TFI 2 (Steel-Dwass test. *P<0.05; **P<0.01; n.s., not significant).

The subjective evaluations of the ocular fundi showed that 57 eyes were categorized as non-tessellated, 27 eyes as weakly tessellated, and 16 eyes as strongly tessellated (Table 2). The inter-rater agreement of the subjective classifications was high with Fleiss kappa = 0.667 (*P*<0.001). In the multiple comparison analysis, for TFI-2, the difference between the non-tessellated group and weakly tessellated group was not significant (*P = *0.24), and there were significant differences between each of the other categories (*P*<0.001). For TFI-1 and TFI-2, there was a significantly difference between each categories (*P*<0.001, Fig. 2).

**Table 2:** Tessellated Fundus Index for each group.

	TFI-1	TFI-2	TFI-3
**Non tessellated group (N = 57)**	0.332±0.038	0.495±0.025	0.165±0.022
**Weakly tessellated group (N = 27)**	0.363±0.043	0.504±0.022	0.183±0.027
**Strongly tessellated group (N = 16)**	0.413±0.047	0.528±0.028	0.219±0.036

TFI; tessellated fundus index.

The values are the averages ± standard deviations.

### Relationship between TFIs and CT

**Figure 3:**
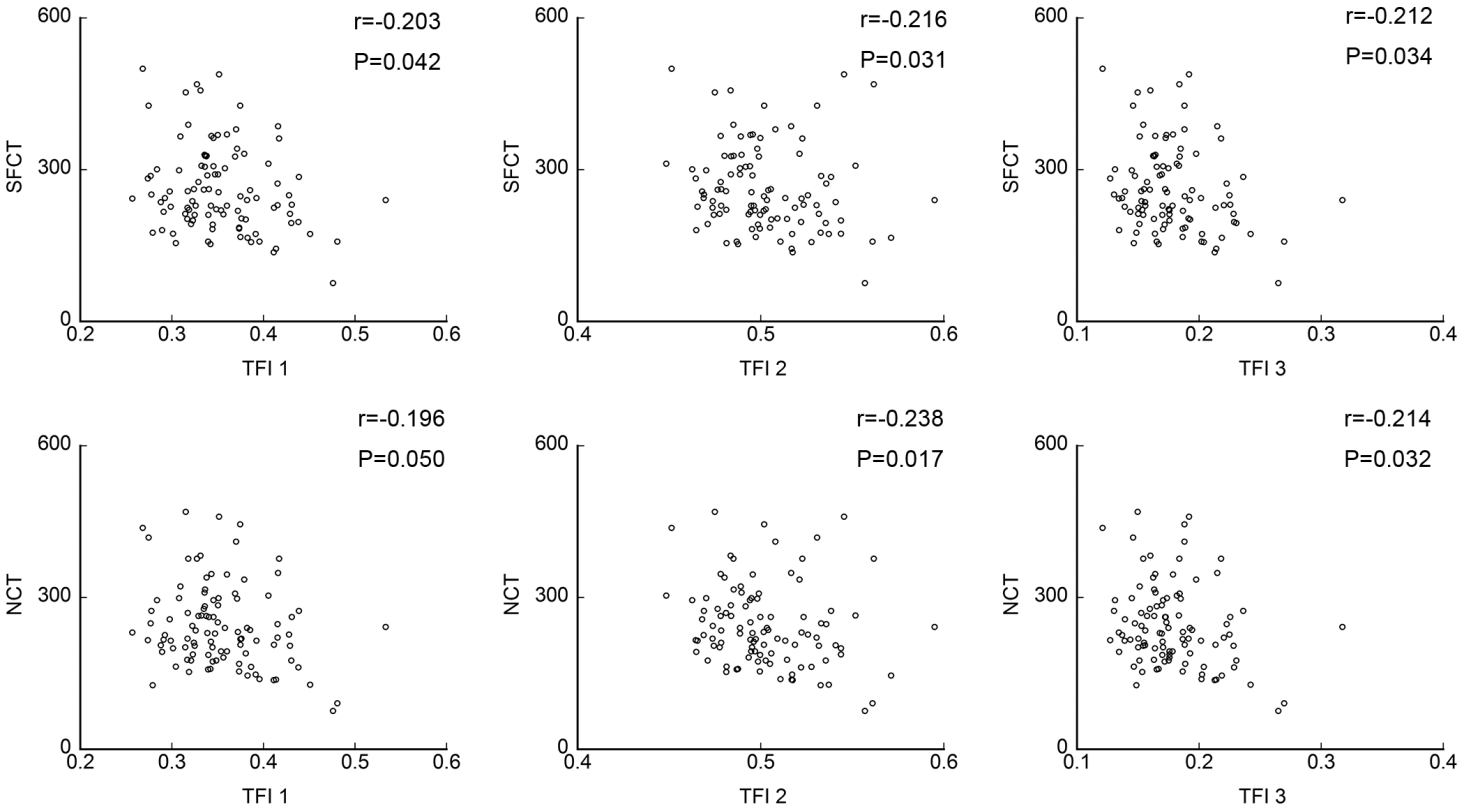
Scatter diagrams of TFIs and subfoveal choroidal thickness (SFCT) and nasal choroidal thickness (NCT). There was a significant and negative correlation between each of the TFIs and both the subfoveal and nasal CTs except for that between TFI-1 and the nasal CT. TFI; tessellated fundus index.

TFI-1 was significantly and negatively correlated with the subfoveal CT (r = −0.203, *P = *0.042), however, it was not significantly correlated with the nasal CT (r = −0.196, *P = *0.050). TFI-2 was significantly and negatively correlated with the subfoveal CT (r = −0.216, *P = *0.031) and the nasal CT (r = −0.238, *P = *0.017). TFI-3 was significantly and negatively correlated with both the subfoveal CT (r = −0.212, *P = *0.034) and the nasal CT (r = −0.214, *P = *0.032; Fig. 3, Table 3).

**Table 3:** Correlation Analysis of TFIs with subfoveal choroidal thickness, nasal choroidal thickness or axial length.

	SFCT		NCT		AL	
	*R*	*P* Value	*R*	*P* Value	*R*	*P* Value
**TFI-1**	−0.203	.042*	−0.196	.050	−0.095	.346
**TFI-2**	−0.216	.031*	−0.238	.017*	−0.196	.051
**TFI-3**	−0.212	.034*	−0.214	.032*	−0.140	.164

*Statistically significant differences among groups (*P*<0.05),

TFI; tessellated fundus index, SFCT; subfoveal choroidal thickness, NCT; nasal choroidal thickness, AL; axial length.

### Relationship between TFIs and axial length

**Figure 4:**
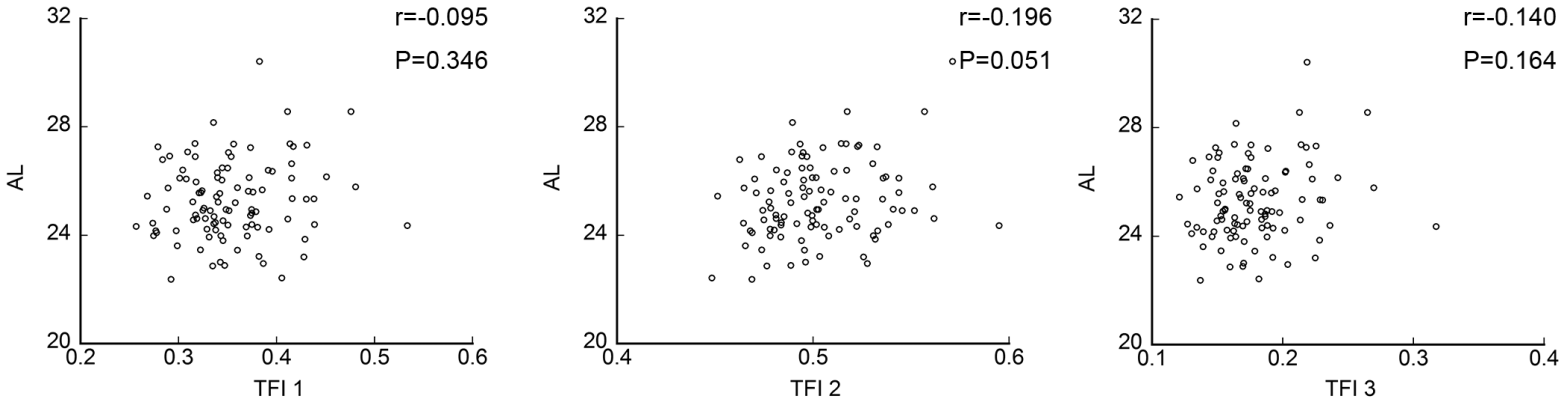
Scatter diagrams of TFI and axial length. No significant correlation was observed between any of the TFIs and axial length. TFI; tessellated fundus index.

**Figure 5:**
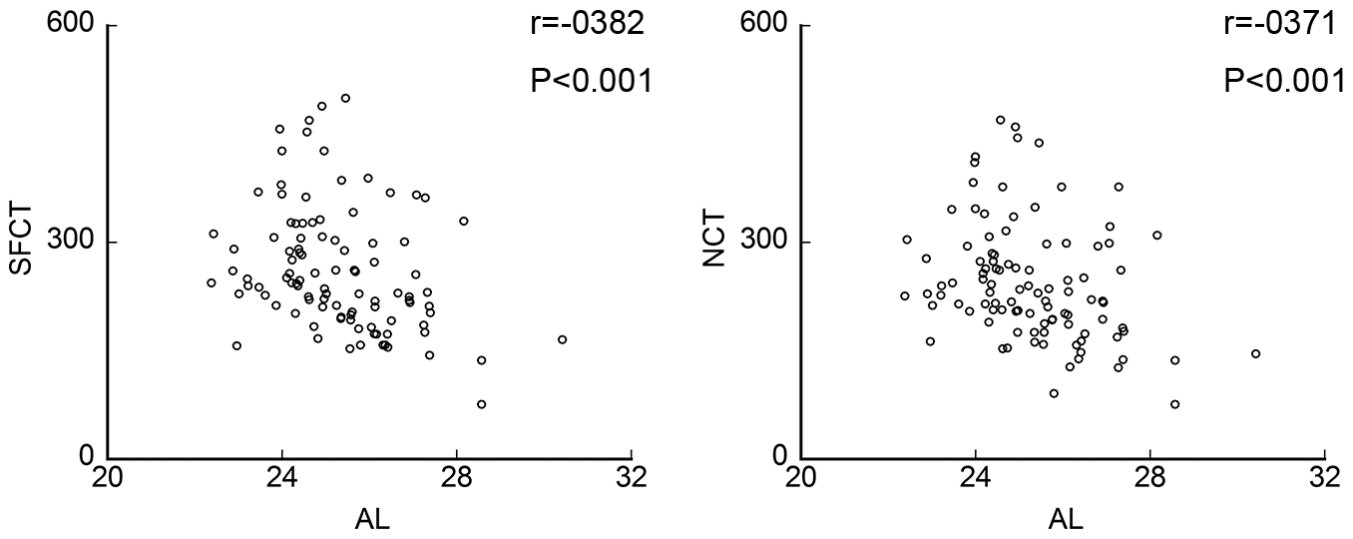
Scatter diagrams of choroidal thickness (CT) and axial length. There was a significant and negative correlation between axial length and both the subfoveal CT and nasal CT.

None of the TFIs was significantly correlated with the axial length (r = −0.095, *P* = 0.346; r = −0.196, *P = *0.051; and r = −0.140, *P = *0.164, respectively, Fig. 4, Table 3). However, the axial length was significantly and negatively correlated with both the subfoveal CT (r = −0.382, *P*<0.001) and the nasal CT (r = −0.371, *P*<0.001**;** Fig. 5, Table 3).

## Discussion

Our results showed that the repeatability of the subjective and objective classifications of the degree of tessellation was high, and that the objective classification with the TFIs was significantly correlated with the subjective classification of the degree of tessellation. Combined, these results indicate that the objective method can be used to determine the degree of tessellation of the fundus of the eye.

Our results also showed that the degree of tessellation was significantly correlated with the CT but not with the axial length. The color of the ocular fundus results from the differential absorption of the observation light by the different components of the retina and choroid as the light passes through and reflected back to the camera. Different pigments in the eye such as the melanin pigment in the RPE and choroid, and the hemoglobin in the blood vessels will absorb different parts of the visible spectrum which will then alter the color of the reflected light [19]. The retina is essentially transparent, and except for the blood in the blood vessels, does not alter the color of the light. Because melanin is black, it does not have a differential absorption of the different wavelengths of the visible spectrum [20]. Thus, the main molecule affecting the color of the fundus is the hemoglobin in the blood vessels in the choroid.

Because the number of RGB pixels in the fundus image determines the index of coloration, the fundus color of a digital image can be calculated using the indices based on the relative RGB pixel numbers [19]. Previously, the hemoglobin in the vessels on the optic nerve head was semi-quantified by the following formulas, R−G, R−B, and (R−G)/R [21]. For our calculations, we used (R-G)/R for TFI-1 because the brightness of the fundus images varied significantly among the images. Suzuki used R/(R+G+B) to quantify the degree of the sunset glow fundus [8]. Using the same formula, Neelum et al evaluated the color of myopic fundi [9]. We also tested this formula for TFI-2, and we also tested the degree of R-G relative to the sum of the R+G+B as TFI-3. The repeatability of the inter-raters and inter-session for the TFIs was very high. The validation of the objective method was made by comparing each TFI with the subjective classification of the tessellation determined ophthalmoscopically. The results showed that all of the TFIs were significantly correlated with the subjective classification of the degree of tessellation.

There were several findings that suggested that TFI-3 was the most suitable index for expressing the degree of tessellation. First, the results of the Steel-Dwass tests showed that TFI-1 and TFI-3 could detect significant differences between any of the three subjective categories, i.e., the non-tessellated group (NT), the weakly tessellated group (WT), and the strongly tessellated group (ST). However, TFI-2 could not detect significant differences between NT and WT. Second, comparing TFI-1 and TFI-3, the *P* values were always lower for TFI-3 than for TFI-1 in detecting the correlation to the choroidal thickness.

Of interest was that the values of TFI were significantly and negatively correlated with the CT. It has been assumed that the tessellated fundus resulted from an increase in the visibility of the large choroidal vessels [22]. This is reasonable because the melanocytes are abundant in the RPE and choroid of normal eyes, and they would block the view of the choroidal vessels. However, the choroid becomes thinner as the axial length elongates, and less melanin pigment would be present between the choroidal vessels and the front of the eye. Because the medium and large vessels of choroid become more visible, it would make the fundus appear more tessellated.

It has been reported that the presence of a tessellated fundus might be an important sign for predicting the progression of the pathological changes of choroid. It was reported that among 276 eyes with a tessellated fundus, 28 eyes (10.1%) progressed to diffuse chorioretinal atrophy, 8 eyes (2.9%) to lacquer cracks, and one eye (0.4%) to choroidal neovascularization after long follow-up periods [6]. Thus the present method would be helpful for examining the future risk of diffuse chorioretinal atrophy, although the current study was conducted using only healthy myopic eyes and a future study should be carried out on patients with pathological myopia.

Although a significant correlation was found between the axial length and the CT in earlier studies [17], [23], the correlation between the TFIs and axial length was not significant. Neelem et al reported that the degree of choroidal atrophy was correlated with the axial length in highly myopic eyes [9]. Wang et al reported that there was a significant correlation between CT and axial length only in mildly myopic eyes and not in highly myopic eyes [14]. If the CT is significantly correlated with the axial length, the degree of tessellation should also be correlated with the axial length. In this study, the CT was significantly correlated with the axial length, however, the degree of tessellation was not significantly correlated with the axial length. The reason for the discrepancy may be because we studied younger eyes without pathologically myopic changes. In healthy myopic eyes, the correlation of axial length and CT is probably more complex.

A severe retinochoroidal degeneration is one of the most serious abnormality associated with high myopia [7]. Although the axial length and CT are associated with retinochoroidal degeneration to some extent, it is not the most decisive factor in predicting these degenerative changes [6], [7]. Rather, the degree of tessellated fundus may be a more important factor. However, the relationship of the degree of tessellation and the retinochoroidal degeneration has not been well studied mainly because of the lack of an objective and quantitative method to evaluate the degree of tessellation. Because the present objective analysis can be used on conventioanl fundus photographs, the present method should enable clinicians to examine a large number of patients for longer follow-up periods easily.

In conclusion, we have developed an objective and quantitative method to determine the degree of tessellation named the TFIs. The results show that the degree of tessellation is significantly correlated with the CT. We believe this method will be of great help to evaluate the ocular fundus objectively and contribute to the understanding of the retinal pathophysiology especially for eyes with myopic changes.

## Supporting Information

Figure S1 **Intersession correlations of TFIs.** Thirty eyes were randomly selected, and the TFIs were determined two times by the same rater (NY). The values were almost perfectly matched between sessions
(TIF)

## References

[1] Ghafour IM, Allan D, Foulds WS (1983) Common causes of blindness and visual handicap in the west of Scotland. Br J Ophthalmol 67: 209–213. 6830738 10.1136/bjo.67.4.209 PMC1040020

[2] Iwase A, Araie M, Tomidokoro A, Yamamoto T, Shimizu H, et al (2006) Prevalence and causes of low vision and blindness in a Japanese adult population: the Tajimi Study. Ophthalmology 113: 1354–1362. 16877074 10.1016/j.ophtha.2006.04.022

[3] Xu L, Wang Y, Li Y, Wang Y, Cui T, et al (2006) Causes of blindness and visual impairment in urban and rural areas in Beijing. The Beijing Eye Study. Ophthalmology 113: 1134–1141. 16647133 10.1016/j.ophtha.2006.01.035

[4] Sawada A, Tomidokoro A, Araie M, Iwase A, Yamamoto T, et al (2008) Refractive errors in an elderly Japanese population: the Tajimi study. Ophthalmology 115: 363–370. 18243904 10.1016/j.ophtha.2007.03.075

[5] Chen SJ, Cheng CY, Li AF, Peng KL, Chou P, et al (2012) Prevalence and associated risk factors of myopic maculopathy in elderly Chinese: the Shihpai eye study. Invest Ophthalmol Vis Sci 53: 4868–4873. 22743322 10.1167/iovs.12-9919

[6] Hayashi K, Ohno-Matsui K, Shimada N, Moriyama M, Kojima A, et al (2010) Long-term pattern of progression of myopic maculopathy: a natural history study. Ophthalmology 117: 1595–1611. 20207005 10.1016/j.ophtha.2009.11.003

[7] Hsiang HW, Ohno-Matsui K, Shimada N, Hayashi K, Moriyama M, et al (2008) Clinical Characteristics of Posterior Staphyloma in Eyes with Pathologic Myopia. Am J Ophthalmol 146: 102–110. 18455142 10.1016/j.ajo.2008.03.010

[8] Suzuki S (1999) Quantitative evaluation of “sunset glow” fundus in Vogt-Koyanagi-Harda disease. Jpn J Ophthalmol 43: 327–333. 10482481 10.1016/s0021-5155(99)00016-7

[9] Neelam K, Chew RY, Kwan MH, Yip CC, Au Eong KG (2012) Quantitative analysis of myopic chorioretinal degeneration using a novel computer software program. Int Ophthalmol 32: 203–209. 22481598 10.1007/s10792-012-9542-4

[10] Spaide RF, Koizumi H, Pozzoni MC (2008) Enhanced depth imaging spectral-domain optical coherence tomography. Am J Ophthalmol 46: 496–500. 10.1016/j.ajo.2008.05.032 18639219

[11] Takahashi A, Ito Y, Iguchi Y, Yasuma TR, Ishikawa K, et al (2012) Axial length increases and related changes in highly myopic normal eyes with myopic complications in fellow eyes. Retina 32: 127–133. 21886022 10.1097/IAE.0b013e318214d094

[12] Chen FK, Yeoh J, Rahman W, Patel PJ, Tufail A, et al (2012) Topographic variation and interocular symmetry of macular choroidal thickness using enhanced depth imaging optical coherence tomography. Invest Ophthalmol Vis Sci 53: 975–985. 22232433 10.1167/iovs.11-8771

[13] Fujiwara T, Imamura Y, Margolis R, Slakter JS, Spaide RF (2009) Enhanced depth imaging optical coherence tomography of the choroid in highly myopic eyes. Am J Ophthalmol 148: 445–450. 19541286 10.1016/j.ajo.2009.04.029

[14] Wang NK, Lai CC, Chu HY, Chen YP, Chen KJ, et al (2012) Classification of early drytype myopic maculopathy with macular choroidal thickness. Am J Ophthalmol 153: 669–677. 22071232 10.1016/j.ajo.2011.08.039

[15] Yamashita T, Asaoka R, Tanaka M, Kii Y, Yamashita T, et al (2013) Relationship between position of peak retinal nerve fiber layer thickness and retinal arteries on sectoral retinal nerve fiber layer thickness. Invest Ophthalmol Vis Sci 54: 5481–5488. 23847316 10.1167/iovs.12-11008

[16] Yamashita T, Tanaka M, Kii Y, Nakao K, Sakamoto T (2013) Association between retinal thickness of 64 sectors in posterior pole determined by optical coherence tomography and axial length and body height. Invest Ophthalmol Vis Sci 54: 7478–7482. 24168996 10.1167/iovs.13-12586

[17] Yamashita T, Yamashita T, Shirasawa M, Arimura N, Terasaki H, et al (2012) Repeatability and reproducibility of subfoveal choroidal thickness in normal eyes of Japanese using different SD-OCT devices. Invest Ophthalmol Vis Sci 53: 1102–1107. 22247474 10.1167/iovs.11-8836

[18] Tan CS, Ouyang Y, Ruiz H, Sadda SR (2012) Diurnal variation of choroidal thickness in normal, healthy subjects. Invest Ophthalmol Vis Sci 53: 261–266. 22167095 10.1167/iovs.11-8782

[19] Orihuela-Espina F, Claridge E, Preece JS (2003) Histological parametric maps of the human ocular fundus: preliminary result. Medical Image Understanding and Analysis: 133–136.

[20] Norman J, Juster NJ (1962) Color and chemical constitution. J Chem Educ 39 *;* 596–601.

[21] Gonzalez de la Rosa M, Gonzalez-Hernandez M, Sigut J, Alayon S, Radcliffe N, et al (2013) Measuring hemoglobin levels in the optic nerve head: comparisons with other structural and functional parameters of glaucoma. Invest Ophthalmol Vis Sci 54: 482–489. 23221075 10.1167/iovs.12-10761

[22] Tokoro T (1998) Types of fundus changes in the posterior pole. In: Tokoro T, ed. Atlas of Posterior Fundus Changes in Pathologic Myopia. Tokyo, Springer-Verlag 5–22.

[23] Flores-Moreno I, Francisco L, Duker JS, Ruiz-Moreno JM (2013) The relationship between axial length and choroidal thickness in eyes with high myopia. Am J Ophthalmol 155: 314–319. 23036569 10.1016/j.ajo.2012.07.015

