# Supplementary figures and images for: Objective Analyses of Tessellated Fundi and Significant Correlation between Degree of Tessellation and Choroidal Thickness in Healthy Eyes
Source: PLoS One. 2014 Jul 28;9(7):e103586. doi: 10.1371/journal.pone.0103586 (PMC4113439; doi:10.1371/journal.pone.0103586)

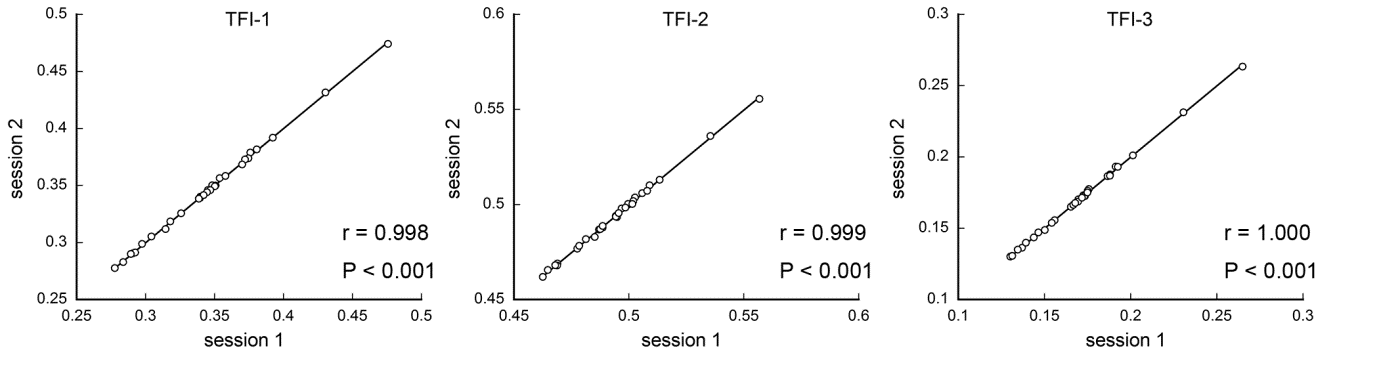

Supplement: Figure S1 — Intersession correlations of TFIs. Thirty eyes were randomly selected, and the TFIs were determined two times by the same rater (NY). The values were almost perfectly matched between sessions (TIF) [file pone.0103586.s001.tif]
